# Association of intracellular and synaptic organization in cochlear inner hair cells revealed by 3D electron microscopy

**DOI:** 10.1242/jcs.170761

**Published:** 2015-07-15

**Authors:** Anwen Bullen, Timothy West, Carolyn Moores, Jonathan Ashmore, Roland A. Fleck, Kirsty MacLellan-Gibson, Andrew Forge

**Affiliations:** 1 Centre for Auditory Research, UCL Ear Institute, London WC1X 8EE, UK; 2 Institute of Structural and Molecular Biology, Birkbeck College, London WC1E 7HX, UK; 3 Neuroscience, Physiology & Pharmacology, UCL, London WC1E 6BT, UK; 4 Centre for Ultrastructural Imaging, King's College London, London WC2R 2LS, UK; 5 National Institute for Biological Standards and Control, Potters Bar EN6 3QG, UK

**Keywords:** Inner hair cell, Intracellular membranes, Synapse, 3D electron microscopy

## Abstract

The ways in which cell architecture is modelled to meet cell function is a poorly understood facet of cell biology. To address this question, we have studied the cytoarchitecture of a cell with highly specialised organisation, the cochlear inner hair cell (IHC), using multiple hierarchies of three-dimensional (3D) electron microscopy analyses. We show that synaptic terminal distribution on the IHC surface correlates with cell shape, and the distribution of a highly organised network of membranes and mitochondria encompassing the infranuclear region of the cell. This network is juxtaposed to a population of small vesicles, which represents a potential new source of neurotransmitter vesicles for replenishment of the synapses. Structural linkages between organelles that underlie this organisation were identified by high-resolution imaging. Taken together, these results describe a cell-encompassing network of membranes and mitochondria present in IHCs that support efficient coding and transmission of auditory signals. Such techniques also have the potential for clarifying functionally specialised cytoarchitecture of other cell types.

## Abstract (precis)

**Summary:** 3D electron microscopy reconstructs the highly organised structure of the infranuclear region of the cochlear inner hair cell, which supports synaptic functions.

## INTRODUCTION

Vertebrate tissues and cells are highly structurally organised. The topographical distribution of molecules, the arrangement of macromolecular assemblies and the disposition of subcellular organelles determine the way in which a cell works. Architectural details are therefore crucial to understanding how cells function and how they are disrupted during disease. Modern 3D electron microscopy techniques have opened new frontiers in the understanding of cytoarchitecture. In this paper, these techniques are used to characterise a highly specialised cell type with a well-defined function, the inner hair cells (IHCs) of the mammalian cochlea, although the techniques are applicable to a wide range of sensory and neural structures where there is a rapid turnover of membrane.

IHCs are the sound-sensing cells present in the organ of Corti, and they convert sound-induced vibrations of the sensory epithelium into electrical signals that are transmitted to the postsynaptic nerve. Each cell is innervated in the infranuclear region (below the nucleus) of the cell by up to 20 afferent nerve fibres (Francis et al., 2004) with a range of sensitivities and spontaneous activity. There is evidence for an organised pattern of fibres with different physiological properties around the circumference of the basal pole of the IHC and along its basal-apical axis (Liberman, 1982; Francis et al., 2004; Liberman et al., 2011). This arrangement implies some further degree of organisation within the cell so that neurotransmitter release and vesicle turnover are directed to occur at the right place and time. This aspect of synaptic organisation is not well understood.

The ability of the IHC synapse to sustain transmission for long periods has been attributed to the presence of the synaptic ribbon, a specialised synaptic structure that is also found in retinal photoreceptors and bipolar cells. Ribbons are anchored near the synapse and surrounded by synaptic vesicles. It is thought that the vesicles provide a ‘readily releasable pool’ that can be quickly recruited at the onset of sound stimulus, and that the ribbon facilitates the supply of vesicles to the synapse during prolonged stimulation (Nouvian et al., 2006; Safieddine et al., 2012). The ribbon has also been implicated in the fusion of vesicles, priming them for release, and in vesicle biogenesis (Matthews and Sterling, 2008; Snellman et al., 2011; Kantardzhieva et al., 2013). Several hypotheses have been suggested for the recycling and replenishment of vesicles at the ribbon during periods of prolonged stimulation, including both the generation of vesicles at or near the synapse, and the generation and transport of vesicles from more distant sites (Heidrych et al., 2009; Neef et al., 2014). These hypotheses require membrane structures to be present in the infranuclear region of IHCs and suggest that such membrane organelles must be precisely organised for effective synaptic transmission.

Intracellular membrane systems in the infranuclear regions of IHCs have been described as ‘canaliculi’ coursing from beneath the nucleus to contact the basal plasma membrane near synapses, and linear cisternae with characteristics of rough endoplasmic reticulum (RER), associated with mitochondria and cytoplasmic vesicles (Spicer et al., 1999). As Golgi bodies have not been observed in the infranuclear region of the IHC, it has been suggested that the vesicles do not carry a protein cargo but might contain neurotransmitter (Spicer et al., 1999, 2007). Membrane cisterns local to the synapse and synaptic ribbon have also been implicated in roles of membrane retrieval and recycling after synaptic stimulation (Lenzi et al., 2002; Kantardzhieva et al., 2013).

We describe here the large-scale reconstruction of the IHC infra-nuclear region and its associated innervation using a combination of 3D electron microscopy techniques, including serial block face scanning electron microscopy (SBF-SEM) (Denk and Horstmann, 2004) and high-resolution electron tomography. By combining these methods it is possible to examine cytoarchitecture from the level of whole-cell shape to macromolecular complexes, encompassing the topographical distribution of cellular contents, and the structure of individual organelles and cytoskeletal elements. Such methods provide detailed structural information contextualised by the wider cytoarchitecture and are potentially applicable to many cell types. Using these techniques, we have dissected how cell shape and complex internal organisation relate to afferent terminal arrangement and synaptic transmission.

## RESULTS

### IHCs are asymmetric and non-uniformly orientated in the organ of Corti

**Fig. 1.**
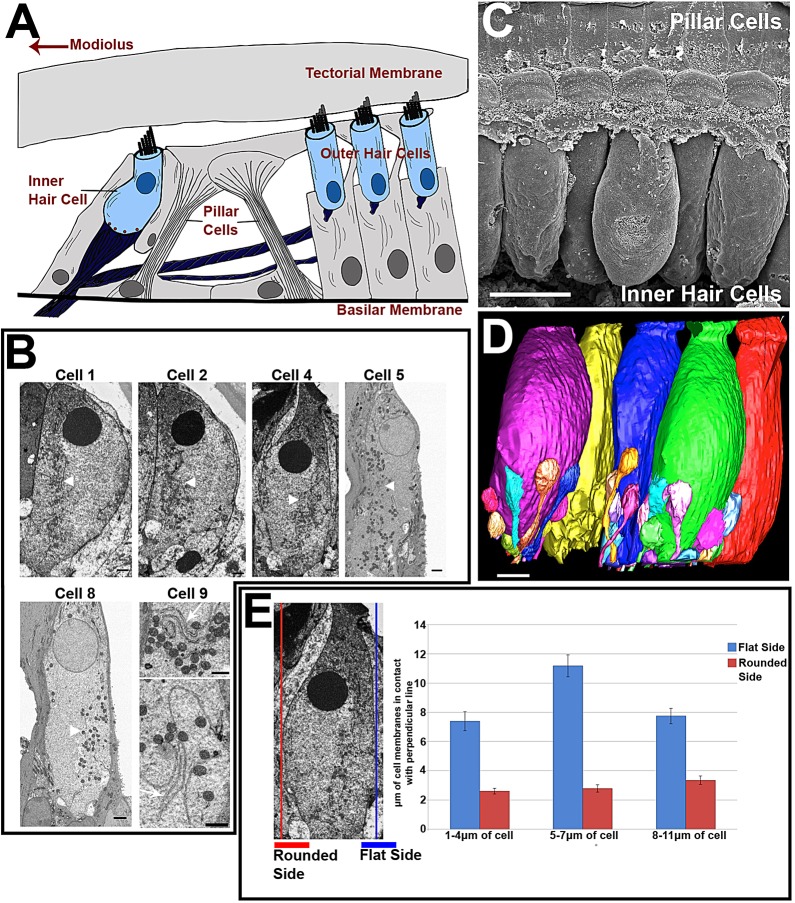
**SBF-SEM analysis of IHCs from the middle cochlear coil of C57/Bl6 mice.** (A) Diagram of the organ of Corti. Direction of modiolus and pillar cells are shown. (B) Whole-cell sections from five hair cells. Concentration of internal membranes and mitochondria on one side of the cell is shown (white arrowheads). Images from cell 9 show the internal lumen of the membranes. (C) SEM micrograph showing IHCs viewed from the modiolar side of the cell. (D) Reconstruction of five IHCs viewed from the same position. Innervation of three of the cells is also reconstructed. Cells are non-uniformly orientated and either the rounded (purple and green) or flattened (yellow, blue and red) side can face the modiolus. (E) Flatness of cells measured by contact of the cell membrane with a perpendicular line. Lines were placed on either side of each cell (*n*=11). Mean±s.e.m. measurements of three regions showed that one side of the cell was consistently flatter than the other. Scale bars: 2 µm (B, cells 1, 2, 4, 5 and 8); 1 µm (B, cell 9); 10 µm (C); 5 µm (D).

IHCs have a characteristic ‘flask’ shape, with a constriction in the neck region. Relative to the surface of the organ of Corti, the cell body is angled towards the centre of the cochlear spiral (the modiolus) and away from the supporting pillar cell (Fig. 1A). The nucleus is located in the apical half of the cell, with the afferent nerve endings forming synapses around the entire baso-lateral region below the level of the nucleus.

For this study, 11 IHCs from the middle cochlear coil of two mice were examined. Fig. 1 shows that the cell bodies were asymmetric in shape, being rounded (bulbous) on one side and flatter on the other (Fig. 1B,E). The orientation of the cell with respect to the rounded side was not consistent: for some IHCs the rounded side faced towards the pillar cell, for others it faced the modiolus. This is evident from SEM images of the row of IHCs in the organ of Corti broken along the pillar cell region (Fig. 1C), but was confirmed by 3D reconstructions of segments of organ of Corti imaged by SBF-SEM (Fig. 1D). The flatness of each side of the IHC was quantified using a perpendicular line placed along each side of the cell. Mean measurements from three regions of the cells (1–4 µm, 5–7 µm and 7–11 µm) showed that there was a consistent flatness on one side of the cell compared to the other. This side was labelled as the ‘flattened’ side of the cell (Fig. 1E).

IHCs consistently showed an extensive and seemingly continuous network of intracellular membranes throughout the infranuclear region. The membranous network was asymmetrically distributed, appearing more extensive on the flattened side of the cell compared to the rounded side (white arrowheads in cells 1, 2, 4, 5 and 8 in in Fig. 1B) regardless of the orientation of the IHC with respect to the modiolus. This distribution appeared particularly distinct in the region surrounding the nucleus. Examination of intracellular membranes (e.g. from cell 9) showed that the intracellular membranes consisted of a membrane enclosing a cisternal space. This is consistent with the structure of endoplasmic reticulum (Fig. 1B, white arrows in cell 9).

### IHC afferent terminals cluster on the flattened side of the cell

To determine whether the asymmetric shape of the IHC was reflected in an asymmetry in the innervation of the cell, the positions of synaptic structures around the IHCs were examined. Only cells where the entire basolateral plasma membrane and IHC innervation were present in the image stack were used for analysis of the positions of neurons and ribbon synapses. Eight cells (1, 2, 5 and 6–10) fulfilled this criterion. Afferent terminals were identified based on the presence of a synaptic density on the afferent ending and corresponding synaptic ribbon.

**Fig. 2.**
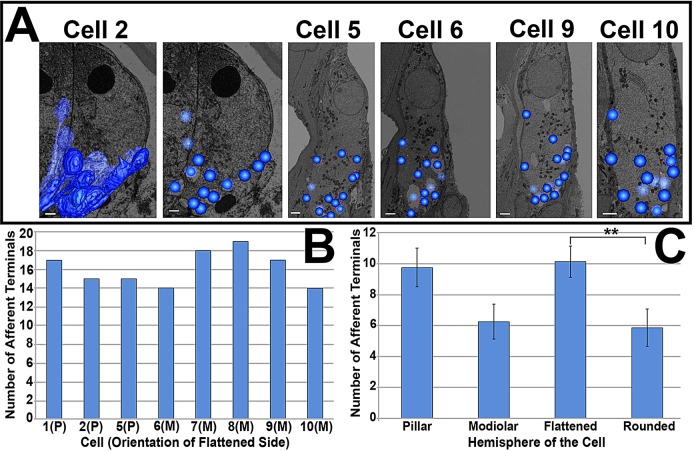
**Distribution of afferent terminals around IHCs.** (A) Distribution of afferent terminals (dark blue) on five cells. Terminals were either manually segmented (cell 2) or represented by a sphere at the approximate centre of the bouton. Comparison between manual segmentation and sphere representation is shown for cell 2. (B) Number of afferent terminals present on each cell studied. (C) Mean±s.e.m. distribution of afferent terminals in the pillar and modiolar, and rounded and flattened hemispheres of *n*=8 cells. **P<0.05. Scale bars, 2 µm. See also supplementary material Table S1.

Each cell had between 14 and 19 afferent terminals (16.1±1.9, mean±s.e.m., *n*=8). There was no correlation between the orientation of the flattened side of the cell on the modiolar-pillar axis of the organ of Corti and the total number of afferent terminals synapsing on the cell (Fig. 2B; supplementary material Table S1). Each cell also had between 14 and 19 synaptic ribbons (16.4±1.9, *n*=8) (supplementary material Table S1). Three of the eight cells had afferent terminals with two associated ribbons; two cells had one double-ribbon synapse and one cell had two double-ribbon synapses.

For 3D reconstruction, the positions of afferent terminals were marked on cells with a representative sphere centred on the approximate centre of the synaptic bouton (cells 2, 5, 6, 9 and 10 in Fig. 2A). In addition, and to provide a more detailed view, the terminals of two cells (cells 1 and 2 in Fig. 6B, and Fig. 2A, respectively) were segmented manually. A comparison between manual segmentation and centred spheres that shows the similarity of these two approaches can be seen in Fig. 2A (cell 2).

Comparison of the modiolar and pillar hemispheres of the cells showed that the mean number of terminals on the pillar side was not significantly different from the number on the modiolar side. In contrast, in seven of the eight cells examined, the majority of afferent terminals were observed on the flattened side of the cell. The mean number of terminals on the flattened side was 10.1±1 compared to 5.9±1.2 on the rounded side, a significant difference (*P*<0.05). The discrepancy between the two sides was largest when the flattened side faced the pillar cell.

It has been suggested that the position of the afferent terminal on the longitudinal axis of the cell (i.e. luminal to basal cell membrane) might also be important in the arrangement of terminals around the IHC (Liberman et al., 2011), so this position was also examined in these cells. In seven of the eight cells examined, the terminal furthest from the synaptic pole on the longitudinal axis of the cell was on the pillar side of the cell, with the only exception being cell 8 (supplementary material Table S1). This relationship was found irrespective of the side of the cell that had the most afferent terminals.

### Intracellular membranes and mitochondria are concentrated on the flattened side of the cell

The concentration of afferent terminals to the flattened side of the cell, coupled with the preferential organisation of membranes and mitochondria on this side suggests a possible relationship between the synaptic sites and intracellular membrane structures. To analyse quantitatively the 3D distribution of intracellular membranes and mitochondria, stereology was used. Stereology is a method that produces statistical information about a 3D object by sampling data (in this case using a grid of points) from 2D sections. Stereological and other 3D analyses were confined here to the infranuclear region of the cell.

Within the infranuclear region, only an ER-like membrane system, mitochondria and synaptic ribbons were evident, but no other cellular organelles were observed. At a magnification that would allow the imaging of whole cells in a single image (voxel size 19×19×50 nm), smaller objects, such as microtubules and membrane vesicles, could not be resolved. Although Golgi bodies were visible in the supranuclear region, they were not observed in the infranuclear portion, which is consistent with previous studies (Spicer et al., 1999).

The distribution of the intracellular membranes and mitochondria in the infranuclear region was examined using a point-counting stereology technique. The two organelles were examined together because, in all of the datasets examined, 95–98% of mitochondria were in close association with strands of intracellular membrane (i.e. with no visible gap between the mitochondrion and membrane strand in section images) (supplementary material Table S1). This showed an almost exclusive association between the two organelles in this region of the IHC, indicating that membranes and mitochondria formed complexes throughout the infranuclear region and were part of the same network.

**Fig. 3.**
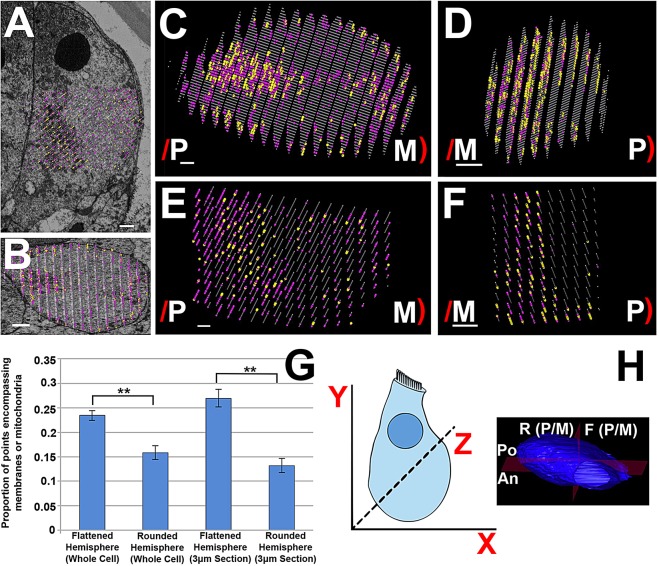
**Distribution of membranes and mitochondria revealed by stereology analysis of IHCs.** (A,B) Stereology grid superimposed on an image of cell 2, showing the longitudinal (A) and radial (B) section. (C,D) Stereology models of whole cells (cell 2 and cell 8). (E,F) 3-µm sections around the nucleus of the same cells. The flattened side of the cell is indicated by ‘/’ and rounded side by ‘)’; P and M indicate pillar and modiolar orientation, respectively. The concentration of membranes and mitochondria (pink and yellow) to the flattened side of the cell is visible. (G) Mean±s.e.m. distribution of membranes and mitochondria between flattened and rounded hemispheres of the IHC in 3-µm sections and whole-cell reconstructions (*n*=9 and *n*=11, respectively). (H) Orientation of the IHC on the *x-*, *y-* and *z*-axes, and orientation of division along the anterior-posterior axis (An and Po) and the rounded-flattened axis (R and F). The sides of this axis can be pillar or modiolar (P/M) depending of the orientation of the cell in the organ of Corti. Scale bars: 2 µm. See also supplementary material Table S1.

To analyse the spatial patterning of the organelles, the whole cell was divided into hemispheres and sampled with stereology point grids at 200-nm intervals. Fig. 3H shows the orientation of cells with respect to the *x*- *y*-* *and *z-*axes, and the division of the cell into hemispheres: the cell was divided into hemispheres through the centre of the cell, along the *y*-*z* plane (denoted ‘rounded’ and ‘flattened’ hemispheres with reference to the cell shape, or denoted ‘modiolar’ and ‘pillar’ hemispheres with reference to the orientation of the cell in the organ of Corti). The cell was also divided along the *x*-*y* plane (‘posterior’ and ‘anterior’ hemispheres). An example of a stereology point grid and its associated micrograph is shown for cell 2 (Fig. 3A,B) in the longitudinal (Fig. 3A) and radial (Fig. 3B) sectioning planes. Points in the cytoplasm (grey), on intracellular membranes (pink) and mitochondria (yellow) are shown. Examples of stereology grids from whole cells are also shown, from cell 2 (Fig. 3C) and from cell 8 (Fig. 3D).

Eight of the nine cells showed membranes and mitochondria enriched in the flattened hemisphere of the cell, regardless of the pillar-modiolar orientation of the flattened side. The SBF-SEM images showed that the asymmetry of intracellular membrane distribution was most prominent in the region of the cell closest to the centre of the nucleus, so a more detailed analysis was also carried out on a 3-µm thick volume of the cell centred around the longitudinal midline of the nucleus that was sampled every 50 nm (i.e. every SBF-SEM section). A further two cells, for which the datasets for whole-cell analysis were incomplete, were included in this analysis. Examples of partial volume grids for cells 2 and 8 are shown in Fig. 3E,F. In all 11 cells analysed, the concentration of membranes and mitochondria was greater in the flattened hemisphere of the cell. The mean results of both analyses are shown in Fig. 3G. The difference in membrane distribution between the flattened and rounded hemispheres of the cell was significant in both the whole-cell and partial cell volume (*P*≤0.01), indicating that membranes and mitochondria segregate asymmetrically towards the flattened side of the cell. The distribution of membranes and mitochondria was also examined in the opposite axis of the IHC, to test whether there was asymmetry in the two hemispheres facing the basal or apical directions along the organ of Corti spiral, by analysing the ‘anterior’ and ‘posterior’ hemispheres (An and Po on Fig. 3H). The proportion of points classified as membranes and mitochondria in the anterior hemisphere (0.21±0.01, mean±s.e.m., *n*=9 cells) was not significantly different to the posterior hemisphere (0.19±0.01). Neither of these hemispheres were significantly different to the results in the flattened hemisphere (0.23±0.01). However, the proportion of points classified as membrane and mitochondria was significantly increased in the anterior hemisphere compared to in the rounded hemisphere (0.16±0.01; *P*<0.05), although the posterior hemisphere and rounded hemisphere showed no significant difference in this number. Taken together, these quantitative results suggest that there is a horseshoe-shaped distribution of the membrane-mitochondria network, which is concentrated towards the flattened hemisphere.

### Common features of mitochondrial distribution

**Fig. 4.**
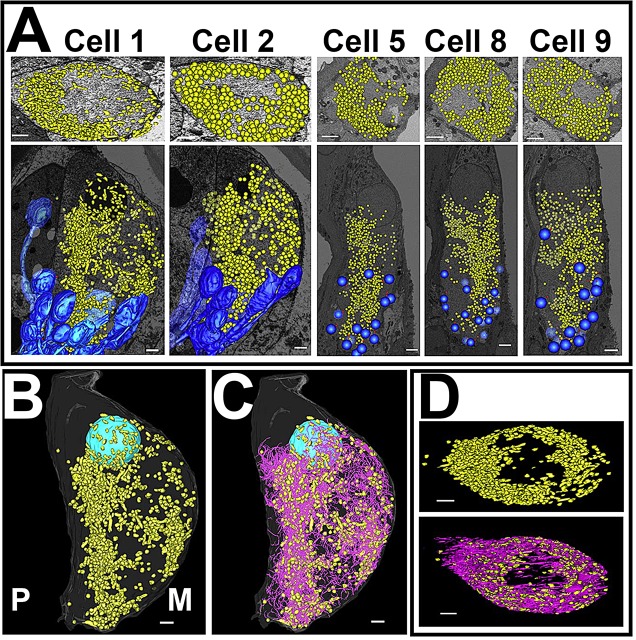
**Reconstruction of mitochondria in IHCs.** (A) Mitochondrial distribution in IHCs, reconstructed using manual segmentation or representative spheres. Top row of images, the view from the nucleus to the middle of the infranuclear region is shown, overlaid on the radial section image of the cell at the centre of the infranuclear portion. Bottom row of images, mitochondrial distribution compared to the positions of the afferent terminal population of each cell (dark blue). (B) IHC mitochondria (yellow) reconstructed by manual segmentation. Orientation of the cell relative to the pillar cell (P) and modiolus (M) is shown on the image. (C) Reconstructed mitochondria shown in conjunction with reconstructed intracellular membranes (pink). (D) Mitochondria alone, and the mitochondria and intracellular membrane population together viewed looking towards the basal pole of the cell from the position of the nucleus. Scale bars: 2 µm. See also supplementary material Table S1.

The arrangement of mitochondria suggested by stereology was confirmed by reconstruction of the entire population of mitochondria in the infranuclear region. For all cells except cell 1, mitochondria were visualised as representative spheres to show their distribution. In cell 1, mitochondria were manually segmented (Fig. 4A). Mitochondria in all cells were arranged in a roughly semi-circular shell distribution, concentrated on the flattened side of the cell and with a sparse central region (Fig. 4A, top row; Fig. 4D), although this region appeared larger in the manually segmented cell than in those where spheres represented mitochondria.

The distribution of mitochondria in the cells was also compared to the distribution of afferent terminals around the cell. These reconstructions showed that the asymmetric distribution of mitochondria matched the distribution of afferent terminals, as was suggested by the above stereology analysis and afferent terminal reconstruction (Fig. 4A, bottom row).

The strong association between membranes and mitochondria (supplementary material Table S1) indicates that mitochondrial reconstruction could be used to estimate the distribution of membrane. This was confirmed by the segmentation of the complete intracellular membrane population in one cell (see next section). Mitochondria (Fig. 1B,C, yellow) and intracellular membranes (Fig. 4C,D, pink) in this cell were manually segmented, and when compared showed almost identical distribution patterns.

### IHC intracellular membrane system can be divided into discrete classes of cisternae

Stereology analyses and the close association of mitochondria with membrane both indicate that intracellular membranes are concentrated on the flattened side of the cell. To probe the details of membrane arrangements, a single cell (cell 1) was chosen from the SBF-SEM stacks for full manual reconstruction of the infranuclear intracellular membranes. The membranes were modelled as tubes, and were designated as parts of a single putative membrane sheet when they lay directly above each other in adjacent SBF-SEM sections (for further details, see Materials and Methods).

**Fig. 5.**
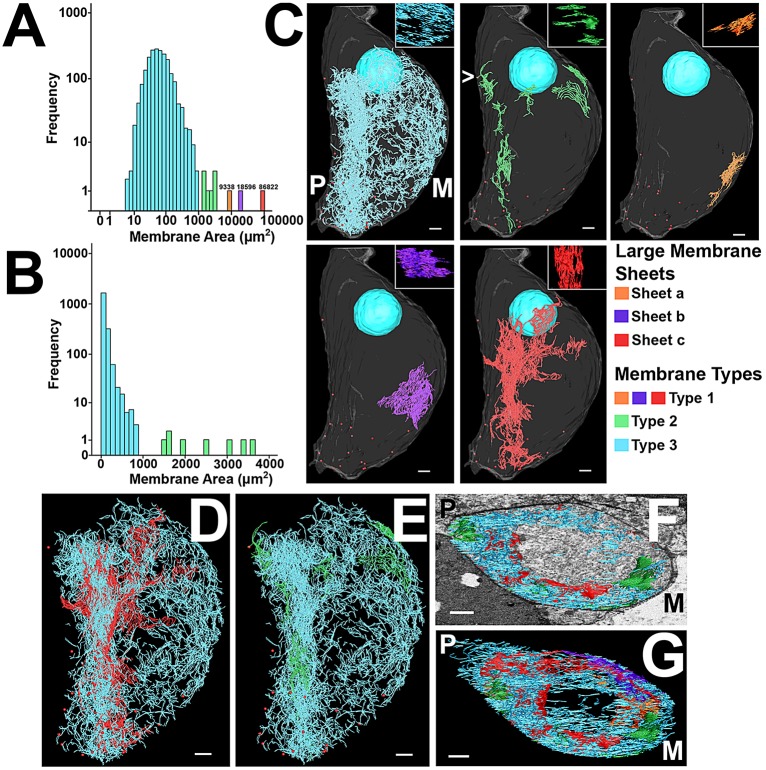
**Reconstruction and classification of IHC intracellular membranes.** (A) Histogram of internal membrane surface areas reconstructed from Cell 1. Sizes of very large membrane sheets are shown above their respective bars. (B) Histogram of intracellular membrane surface area, excluding very large membrane sheets. (C) Membranes are shown divided into the clusters shown in the histogram. Orientation of the cell to the pillar cell (P) and modiolus (M) is shown on images. Red spheres represent positions of ribbon synapses. Insets: membranes aligned perpendicular to the *z*-axis, showing their sheet characteristics. Only a portion of sheet c (red) is shown. >, a Type 2 membrane sheet close to a ribbon. (D) Type 3 (blue) membranes and sheet c (red) are shown together. (E) Type 2 and Type 3 membranes are shown together. (F) Top section of intracellular membrane shown from the position of the nucleus, and with a radial section image. (G) View through the complete length of the cell from the same position. Scale bars: 2 µm. See also supplementary material Table S2 and Movie 1.

Fig. 5A shows a frequency histogram constructed from the estimated surface area measurements of these putative membrane sheets, showing the distribution of intracellular membrane sizes in the infranuclear region. Analysis suggested that membranes could be divided into three classes. Three very large membrane sheets were present in the sample [sheets a (orange), b (purple) and c (red) in Fig. 5], which were collectively named as Type 1 membranes. These membrane sheets together comprised 36.9% of the total intracellular membrane, of which 27.9% was a single sheet, sheet c, which was positioned on the flattened side of the cell and traversed the cell from the nuclear region to the base (red in Fig. 5C). The sheets, both in context with the cell body and ribbon synapse, and aligned perpendicular to the *z*-plane to show their continuity (insets), can be seen in Fig. 5C. These sheets were not continuous with each other or any of the other membranes, and appeared to exist as separate large membrane surfaces.

Two other clusters of membrane were also present. The histogram of membrane areas shows that they might form a single population (blue and green, Fig. 5A). To examine these clusters in more detail, the histogram was re-plotted without the Type 1 membranes (Fig. 5B). This histogram suggests that the smaller membranes form part of a single distribution, but that they can be divided into a population of a large number of very small membranes (the steeply curved region) and a small population of intermediate sized membrane sheets (the flat portion of the curve). The eight separate membrane sheets that formed the flat portion of the distribution were named Type 2 (green). The smallest separate membrane sheets, which formed the steeply curving part of the distribution, were designated Type 3 membranes (blue). Type 3 membranes comprised by far the majority of membrane sheets in the cell, containing 2073 of the total 2084 membrane sheets segmented (57% of the total membrane area). The mean±s.e.m. membrane area for this type was 85±2 µm^2^. Type 2 membranes had a mean area of 2383±305 µm^2^ and comprised 6.1% of the total membrane area (insets Fig. 5C).

The majority of mitochondria in the cell (68%) were associated with a single membrane type, whereas the rest were associated with membranes from two different types. To compare the distribution of mitochondria between different membrane types, only mitochondria associated with a single membrane type were considered. Type 3 membranes, despite comprising the largest total surface area, had the lowest density of mitochondria, at 1.5×10^−3^ per µm^−2^. Type 2 membranes had a density of 2.5×10^−3^ per µm^−2^, and Type 1 membranes a density of 3.9×10^−3^ per µm^−2^. Measurements from the intracellular membrane model are collated in supplementary material Table S2.

Examination of membrane distribution throughout the infranuclear region confirmed the asymmetry shown above, in the stereology analysis. Type 2 and 3 membranes were found around the cell, but both types were concentrated towards the flattened side (Fig. 5C). The position of the large Type 1 sheet c at the flattened side of the cell also contributed a large proportion of membrane. The region occupied by sheet c enclosed 36.9% of the infranuclear cytoplasmic volume, but this volume contained 68.8% of the total surface area of intracellular membranes.

Type 3 membranes were almost always the most peripheral of the membranes on the flattened side. The majority of the Type 1 sheet c was further back from the plasma membrane, behind Type 3 membrane sheets (Fig. 5D). Some Type 2 membrane sheets approached the cell membrane (Fig. 5E). On the rounded side of the cell, the two large Type 1 membrane sheets, sheets a and b, were close to the plasma membrane, but not close to the synaptic ribbons (Fig. 5C). Views of the cell looking along the cell body towards the basolateral pole from the position of the nucleus suggested that Type 3 membranes were the closest membrane type to the cell membrane around the majority of the cell. This angle also showed that there was a concentration of membrane towards the periphery of the cell, sparing the central section, similar to the arrangement previously observed in the mitochondrial reconstructions (Fig. 5F,G).

Of the 19 ribbons in the segmented cell, the closest intracellular membrane sheets of 18 of the ribbons were Type 3 membranes. One ribbon had a proximate Type 2 membrane sheet (marked by ‘>’ in the top middle panel of Fig. 5C). The distances from the closest point of the nearest membrane sheet (of each type) to the approximate centre of each ribbon were calculated – for Type 3, the mean±s.e.m. distance was 515±85 nm, for Type 2 it was 2944±511 nm, and for Type 1 it was 1714±235 nm (*n*=19 for all). Type 3 membranes were significantly closer to the ribbons compared to the other two groups (*P*<0.05), but the difference in distance between Type 2 and Type 1 membranes was not significant. Kantardzhieva et al. (2013) have shown that membrane cisterns are mostly absent near the ribbons, but that the number of cisterns increased with increasing distance from the ribbon, reaching an asymptote at ∼350 nm and dropping off after 800 nm. This seems to agree with 515 nm as being the mean distance from the ribbon to the Type 3 intracellular membranes.

**Fig. 6.**
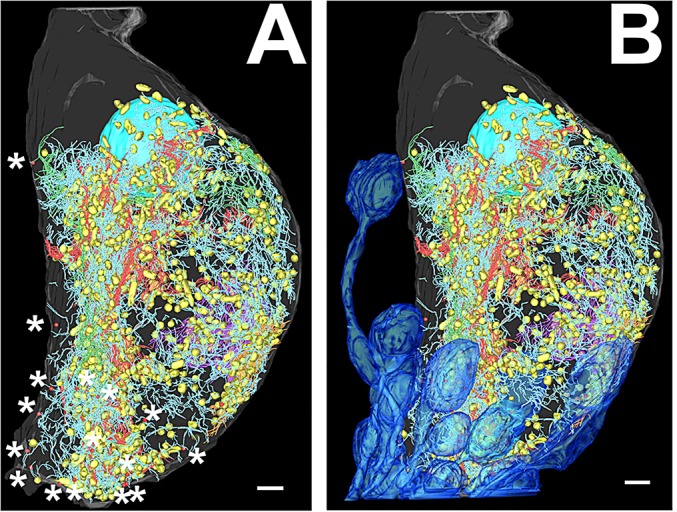
**Complete model of membranes, mitochondria and synapse distribution in cell 1.** (A) Ribbon synapses (red spheres) are marked with asterisks for clarity. (B) Reconstructions of synaptic terminals are shown (blue). Scale bars: 2 µm. See supplementary material Movie 1.

By examining a fully reconstructed cell, it was possible to understand the distribution of the intracellular membrane system in the context of the complete infranuclear region (Fig. 6A,B). Intracellular membranes and mitochondria were concentrated in the regions of afferent terminal boutons, mostly at the base of the cell on the flattened side. The gradient of membrane sizes could also be appreciated, with the smallest Type 3 membranes (blue) closest to the ribbons (* in Fig. 6A), filling the gap between the large membrane sheet and the cell membrane on the flattened side of the cell.

### Filamentous linkages between mitochondria and ER membranes

The colocalisation of membranes and mitochondria seen in the infranuclear region of IHCs prompted a closer examination of the structural relationship of the two organelles in these cells. We therefore used high-resolution electron tomography to examine junctions between intracellular membranes and mitochondria.

**Fig. 7.**
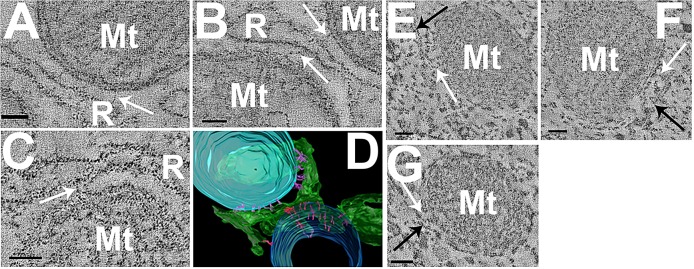
**Tomographic reconstruction of linkages between RER and mitochondria.** (A–C) Slices from tomographic reconstructions of conventionally fixed mouse tissue showing linkages of three different mitochondria (Mt) to RER (R) (white arrows). (D) 3D reconstruction of mitochondria (blue) and membrane (green) showing the depth and arrangement of mitochondria–membrane links (purple). (E–G) Membrane–mitochondria links observed in HPF tissue from a guinea pig. Links (white arrows) are shown between mitochondria (M) and membranes (black arrows). Images E–G show links in an IHC. Scale bars: 50 nm. See supplementary material Movie 2.

**Fig. 8.**
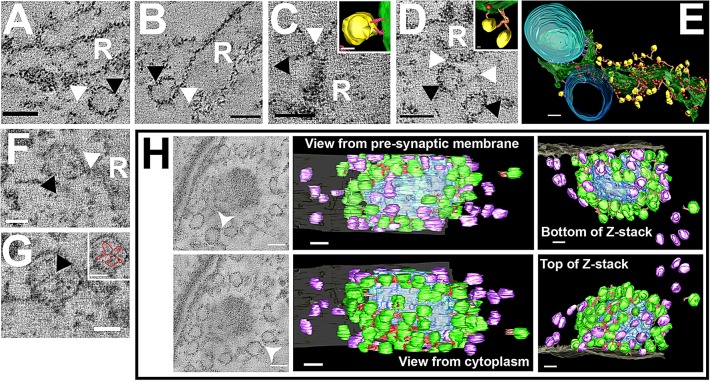
**Tomographic reconstruction of vesicle–membrane and vesicle–vesicle links on ER and at the ribbon synapse.** (A–D) Linkages (white arrowheads) are shown between membrane vesicles (black arrowheads) and three different areas of the RER (R). Images show an average of five tomographic slices. Insets in C and D show reconstruction of these links. (E) Reconstruction of a section of RER (green with ribosomes in red) with linkages to mitochondria (blue) and vesicles (yellow) showing the linkages surrounding the membrane. (F,G) Tomographic reconstructions of vesicles in HPF guinea pig tissue. Images show an average of five tomographic slices. (F) Vesicle membrane linkages (G) HPF prepared vesicle showing apparent internal vesicle structure (black arrow). The inset shows the vesicle internal structure outlined in red. (H) Tomographic reconstruction of a mouse ribbon synapse; images show an average of nine consecutive tomographic slices. The 3D model shows vesicles (green) with vesicle–vesicle links (red) and vesicles without linkages (purple) around the synaptic ribbon (blue). The synapse is viewed from four positions: through the pre-synaptic membrane (grey), from the IHC cytoplasm, and from the top and bottom of the synaptic ribbon. Scale bars: 50 nm (A–D, H); 10 nm (inset in C), 20 nm (inset in D), 100 nm (E), 25 nm (F), 25 nm (G, inset in G). See supplementary material Movie 2.

In image slices from electron tomograms of conventionally fixed mouse tissue, structural linkages between mitochondria and intracellular membrane in the infranuclear region were clearly visible (Fig. 7A–C). The membrane had a clear cisternal space and was studded with electron-dense structures with a diameter of ∼20 nm, similar to the diameter of ribosomes (Zelena, 1972). This membrane was categorised as rough endoplasmic reticulum (RER). Sampling from several sites around the IHC revealed distinct links between mitochondria and the membrane sheet. Reconstruction of these linkages showed them to be extensive, occurring along the profile of a mitochondrion and its interface with the RER membrane (Fig. 7D and Fig. 8E). They had a mean±s.e.m. length of 30.9±1.3 nm (*n*=44), similar to the RER–mitochondrial links described in other cell types (Rowland and Voeltz, 2012). These linkages occurred in regions of the RER that excluded ribosomes (Fig. 8E; supplementary material Movie 2), suggesting a specialised ER domain as seen in other cells (Csordas et al., 2006; Rowland and Voeltz, 2012).

To investigate whether the linkages observed were the result of artefacts arising from glutaraldehyde fixation, additional samples that had been processed by high-pressure freezing (HPF) and freeze substitution were examined. Owing to the requirement to dissect the sensory epithelia clearly from any bony constituents, these samples were obtained from adult guinea pigs, which also allowed for cross species comparisons. Tomograms from these samples showed mitochondrial membrane links (Fig. 7E–G) of similar lengths to those observed in the conventionally fixed samples (mean length 27.5±4.8 nm, *n*=10) and also occurred in regions of membrane that were devoid of ribosomes.

### Small vesicles are linked to ER membranes and to each other at the ribbon synapse

The tomography of mitochondria and RER, revealed a second, unsuspected feature of IHC RER: small membrane vesicles clustered around the RER membranes. Some of these vesicles appeared tethered to the membrane sheet and to each other by thin linkages (Fig. 8A–D). Guinea pig samples prepared by HPF also showed the same vesicle-membrane linkages (Fig. 8F). When these linkages were reconstructed, they showed tethered vesicles along the length of the endoplasmic reticulum. The tethers appeared to connect to the membrane surface, and occasionally to ribosomes and had a mean±s.e.m. length of 52.9±3.3 nm (*n*=63) (Fig. 8C–E; supplementary material Movie 2). Vesicles were also observed tethered to each other away from membranes (data not shown). All membrane sheets examined showed these linkages, including those not directly adjacent to synapses. The membrane vesicles observed had a mean diameter of 37.3±0.6 nm (*n*=70). This value was not significantly different from that of the vesicles at the ribbon synapse segmented by the same method (Fig. 8H) (mean diameter, 36.3±0.5 nm, *n*=89). Linkages between the vesicles and the ribbon had a mean length of 26.8±1.8 nm (*n*=36) and were significantly shorter than the links between vesicles and RER membrane (*P*<0.05). In the HPF samples, vesicles were occasionally observed with internal structure, which appeared as arms radiating from a central point (Fig. 8G).

Linkages between vesicles were also observed at the synaptic ribbon (white arrows in Fig. 8H, shown in red in reconstructions). Compared to the linkages between vesicles at the RER, which had a mean length of 45.4±3.0 nm (*n*=61), links between vesicles at the ribbon were shorter (mean length, 22.3±0.7 nm, *n*=78). Links at the ribbon were significantly shorter than both those between individual vesicles and those between vesicles and RER (*P*<0.05). Both linked and unlinked vesicles were observed at the ribbon synapse (green and purple, respectively, in Fig. 8F and supplementary material Movie 2). Vesicles around the surface of the ribbon that faced the cytoplasm were linked together, but those facing the synaptic gap appeared to have lost these linkages. Vesicles with apparently unattached tethers were also noted around the ribbons, although because the entire vesicle population of unattached vesicles surrounding the ribbon could not be segmented (owing to section thickness and magnification required to resolve the tethers), it was difficult to tell what proportion of these vesicles had unattached tethers.

Taken together, our data demonstrates a high degree of organisation of membranes and of the associated organelles proximal to the synapses, both in overall distribution and in local connectivity, which has potential roles in supporting efficient synaptic transmission.

## DISCUSSION

Specialisation of a cell is likely to involve not just specific structures, but also tailoring of the complete cytoarchitecture to the task in question. The IHC has evolved as a dedicated sensory cell with an extraordinary capability for rapid and sustained neurotransmission of a complex signal, and despite a good understanding of how component parts of the IHC function, wider questions of how the whole cell is tailored to its specialised task have not been comprehensively addressed. By examining the subcellular structures of IHCs in three dimensions, and in context with surrounding cellular and extracellular structures, this study has shown a previously unappreciated level of internal organisation in IHCs and suggested how such organisation might be related to the function of the cell. It seems likely that such cellular organisation underpins specific function in multiple cell types and the methods used here could usefully be applied elsewhere.

### Inner hair cell asymmetry

We have shown that the IHC is an asymmetric cell, and non-uniformly orientated in the organ of Corti. This non-uniform orientation might arise simply as a requirement to increase the packing density for IHCs. It has been shown previously, from confocal imaging studies, that the basolateral poles of adjacent IHCs are often staggered in adjacent radial planes (Yin et al., 2014), and we suggest that this pattern might be a result of the changing of the orientation of the flattened and rounded sides. The intracellular structure of the cell and the pattern of innervation appear to be affected by the asymmetry of the cell, with both intracellular components and afferent terminals distributed preferentially to the flattened side of the cell regardless of the orientation of the cell. Two alternate hypotheses can be proposed from these results: either factors internal to the cell affect afferent patterning, or afferent patterning affects cell shape and internal arrangement. A developmental study would be required to untangle the precise nature of this association but is beyond the scope of the current work.

The complete 3D reconstruction of an IHC infranuclear region suggested that the IHC of the mature mouse can be divided into two topographical regions: (1) a supranuclear ‘synthetic’ pole that contains the cells machinery for protein synthesis in the form of Golgi bodies and endoplasmic reticulum; and (2) an infranuclear ‘synaptic’ pole that contains the ribbon synapses, and a network of endoplasmic reticulum, mitochondria and vesicles. This network is asymmetrically distributed and traverses the cell from the nucleus to the cell base. ER is known to associate with microtubules, with the microtubules acting as cell organisers (Tuffy and Planey, 2012). Although microtubules could not be detected at the resolution of the present SBF-SEM data, it is likely that they were also present. Microtubule tracks in IHCs have been described previously as being orientated longitudinally between the apex and base of the cell (Steyger et al., 1989), suggesting that they follow similar tracks to those described by the membranes in this study.

### Membrane organisation in the IHC

The membrane system reconstructed in this study is most likely a snapshot of a dynamic system that changes in response to the differing energetic demands of the cell, particularly during periods of sustained neurotransmission. Membrane size distributions might represent a sheet or network of membranes that is constantly breaking and reforming. Spicer and co-workers (1999, 2007) have described cisternae that are broken into segments at the sites of mitochondrial contact. This is also sometimes observed in the SBF-SEM data. In the results, we show three distinct categories of membrane sheet. We hypothesise, therefore, that the membrane system is composed of numerous small elements (Type 3 membranes) derived from the larger continuous sheets that make up the Type 1 membranes. Type 2 membranes might represent a transitional form between Type 1 and 3 membrane classes. The relative positioning of the numerous small membrane sheets (‘Type 3’ membranes) and the Type 1 sheet c on the flattened side might also be representative of the edges of a larger membrane sheet (‘Type 1’) breaking into smaller Type 3 membranes, which then project towards the synaptic ribbons. It is also tempting to consider whether the core of the Type 1 membrane sheets represent stable structures. The stability of sheet c might help to explain the consistent concentration of membrane to the flattened side of the cell, and the higher mitochondrial density of the Type 1 membranes.

Membrane cisterns in close proximity to the ribbon might be a consequence of exocytosis, that is, they could be formed by fusion of vesicles during repetitive stimulation. Membrane cisterns close to ribbons have been shown to increase in size upon depletion of synaptic vesicles by stimulation, and it has been proposed that they represented an important component of the vesicle recycling pathway, with vesicles reforming at the cisterns (Lenzi et al., 2002; Kantardzhieva et al., 2013). Cisterns themselves might also be competent for fusion and release of neurotransmitter (Matthews and Sterling, 2008). Although the width of the lumen of the most peripheral intracellular membrane elements described here appears larger than that of the cisterns described by those authors, it is tempting to speculate that they are the same structure. Ribbons have also been shown to attract membrane cisterns, regardless of whether the ribbon is tethered at the synapse or free floating in the cytosol (Khimich et al., 2005). Cytosolic ribbons might be attracting small membrane cisterns such as (or derived from) the ones described in this study, suggesting an attraction for cisterns that are not wholly formed by recycled vesicles. Therefore the membrane network might be altered by the stimulus state of the cell. The distribution (particularly of Type 3 membranes) in the stimulated cell is a target for future work.

### Mitochondrial organisation in IHCs

The results of the electron tomography indicate strongly that the mitochondria-membrane structures observed represent an integrated functional complex. The linkages observed were not artefacts of chemical fixation, and were present in both species examined. Linkages between mitochondria and ER in other cell types have multiple functions, including control of lipid biosynthesis, mitochondrial division, Ca^2+^ signalling and co-ordinating organelle movement (Rowland and Voeltz, 2012). In the IHC, Ca^2+^ regulation in particular seems a likely linkage function, as Ca^2+^ controls the release of neurotransmitter at IHC synapses and intracellular Ca^2+^ levels might be particularly important in sustained signalling. Mitochondria are known to act as sequesters of Ca^2+^ and to control Ca^2+^-meditated neurotransmitter release in some cells (Billups and Forsythe, 2002). Work has also shown that during aminoglycoside-induced hair cell death in zebrafish, elevated intracellular Ca^2+^ occurs immediately after mitochondrial potential collapse (Esterberg et al., 2013). The large cellular scale of single sheets in the membrane-mitochondrial network, the positioning of the sheets around the periphery of the cell and their concentration in regions where the majority of synapses are located suggests that the network might act by controlling Ca^2+^ levels in regions local to the synapses and shuttling Ca^2+^ throughout the cell. Glutamate synthesis might also be a mitochondrial function in IHCs: the enzyme phosphate-activated glutaminase, a key enzyme in the synthesis of glutamate, has been shown to be restricted mainly to the mitochondria and to colocalise with glutamine and glutamate (Takumi et al., 1999). It is possible therefore that the mitochondria in the infranuclear region are also contributing to glutamate recycling there.

### Vesicle tethering in IHCs

The tethering of small vesicles to RER was an unexpected finding, revealed only by high-resolution electron tomography. RER is often associated with transport vesicles for the shuttling of synthesis products between the RER and Golgi complex, but such vesicles are generally larger (50–100 nm in diameter; Martínez-Menárguez, 2013) than those observed here tethered to the RER (∼36 nm). In addition, the Golgi complex was not observed in the region of the infranuclear membrane network, and vesicles were not observed either budding or fusing with the RER membrane, only connected to it by filamentous linkages. The vesicles observed were of the same diameter as putative neurotransmitter vesicles that were associated with or in proximity to ribbon synapse. Immunostaining for the vesicle glutamate uptake transporter VGlut3 in IHCs shows labelling throughout the hair cell cytoplasm (Peng et al., 2013; Neef et al., 2014), which might indicate that neurotransmitter vesicles are also present throughout the cytoplasm and not just concentrated at synapses. If the vesicles do contain neurotransmitter, it is possible that the membrane network might also represent a secondary store of neurotransmitter vesicles for release during sustained synaptic transmission. Ca^2+^-sensing proteins (such as otoferlin) and their binding partners appear to be present throughout the IHC cytoplasm (Duncker et al., 2013; Neef et al., 2014), suggesting they might also be involved in such a system, possibly as constituents of the linkages. It has recently been suggested that otoferlin could be part of the vesicle-priming machinery in the IHC because otoferlin deletion lengthens the linkages and hence could be involved in the production of close attachment of vesicles to the synaptic active zone (Vogl et al., 2015). Thus co-ordination of a secondary vesicle store, vesicle biosynthesis and the putative Ca^2+^-regulating functions of the RER and mitochondria would produce a multifunctional complex capable of regulating a proportion of the vesicle supply to the synapse and the release of neurotransmitter.

The presence of linkages between vesicles at the ribbon synapse raises questions about the putative role such linkages may have in synaptic transmission. At frog neuromuscular junctions it has been shown that linkages and internal vesicle structures might be important in the attachment of vesicles to a docking complex and that they might confer a preferred conformation on docking vesicles. The linkages described in this study might be analogous to the filamentous linkages described at the neuromuscular junction, which link vesicles together and to structures at the synaptic active zone (Szule et al., 2012). It has been reported that internal vesicle structure is not visible after conventional fixation and room temperature staining, but only after rapid freezing and freeze substitution (Harlow et al., 2013). Harlow et al. described the internal vesicle assembly as a bilateral structure with irregular arms radiating from the centre, which is remarkably similar to the internal structure seen in vesicles in our study. A comprehensive study of vesicles in rapidly frozen samples would be required to confirm this result, but it opens an interesting avenue of further investigation into whether the internal structure observed in the neuromuscular junction is also present in the IHC and other sensory cells and whether such linkages perform a similar function in regulating the vesicle-docking conformation.

The cells of the vertebrate cochlea provide an ideal model system in which to evaluate the functional context of distinct populations of highly specialised cells. By combining 3D microscopy analyses, it has been possible to examine and relate cellular organisation at multiple resolution levels in a specialised sensory cell. For the IHC, these observations might have significant future value in determining the changes to the structures inside cells that occur after cochlea damage, for example by excessive noise or ageing. Cochleae exposed to certain noise traumas undergo selective deafferentation of IHCs, and are thought to model so called ‘hidden hearing loss’ in humans (Kujawa and Liberman, 2009). Our preliminary work on cochleae exposed to such noise traumas suggests that the patterns of cellular arrangement described here might be disrupted in noise damaged cochleae. Early analysis of IHCs from noise-damaged animals suggests a breakdown in the organisation of membranes and mitochondria within the cells, coinciding with degenerated afferent terminals and increased numbers of abnormal mitochondrial structures. High-resolution analysis of the regions surrounding degenerated synapses, which we are currently undertaking, also suggests that there are changes to structures local to the synapse, particularly the small membrane structures, indicating possible cellular rearrangements in response to synaptic changes. These results indicate the utility of these approaches in assessing cellular damage.

### Conclusion

3D microscopy approaches and reconstruction on cellular scale have clear applications in identifying the structural specialisations in a wide variety of other cell types. As our understanding of components of cellular systems improve, it is essential to determine how they interact with each other within the system as a whole. Multiscale resolution of cellular structures provides the vital information necessary to understand the particular ways in which cells are organised to perform specific functions, and how disturbances of such organisation underlies the progression of damage ageing and disease.

## MATERIALS AND METHODS

Original data contained in this paper can be accessed by contacting the corresponding author.

### Animals

Cochleae were obtained from two 3-month-old C57/Bl6 mice and one adult tri-colour guinea pig. All work with animals was conducted in accordance with procedures licensed by the British Home Office and approved by the UCL Animal Ethics committee.

### Conventional fixation

Auditory bullae were isolated from mice immediately after death. Cochleae were fixed in 2.5% glutaraldehyde and decalcified in 4% ethylenediaminetetraacetic acid (EDTA) (Sigma, UK). Post fixation staining was carried out in 1% aqueous osmium tetroxide (Agar Scientific, UK) and samples were embedded into Epon resin (TAAB, UK).

### Cryo-fixation and freeze substitution

After death, auditory bullae of the guinea pig were removed and placed on ice. Cochlear tissue was dissected from the bullae just prior to freezing. The time between death and freezing was ∼30 min. Samples were prepared by high-pressure freezing and freeze substitution as previously described (Bullen et al., 2014).

### Serial block face scanning electron microscopy

After fixation and decalcification cochleae were transferred to 0.1% tannic acid (TAAB, UK) in 0.1 M cacodylate and incubated in this solution overnight at 4°C. Samples were processed for SBF-SEM following the method of Deerinck et al. (2010). After staining, samples were dehydrated and embedded in Epon resin. Samples were examined in the Gatan 3View system (Gatan, USA) on a Zeiss Sigma FE-SEM (Zeiss, Germany), or a Gatan 3View XP system on a JEOL 7100F SEM (JEOL UK, UK). Blocks were cut at 50 nm, and the exposed block face imaged with a pixel size of 19 nm.

### Electron tomography

200-nm sections of organ of Corti were examined in a JEOL 2100 transmission electron microscope operating at 200 kV (JEOL) and dual-axis images were collected between at least 60° and −60° at 1° increments. Data collection was controlled by SerialEM software (Mastronarde, 2003). Tomographic reconstruction was carried out by weighted back projection using the IMOD suite of programs (Mastronarde, 1997).

### Segmentation, stereology and modelling

Segmentation, stereology and modelling were carried out using IMOD programs. For modelling of membranes in SBF-SEM images, a fixed threshold level that excluded cytoplasmic background was used. Point-counting stereology was carried out in IMOD. Each grid covered 11.4 µm of the length of the cell (*y*-axis) and the complete width (up to 15.3 µm) of the cell (*x*-axis) (Fig. 2L). More than 5000 points were classified for each stereology data set. For whole-cell measurements, grids were placed 200 nm apart (*z*-axis). Two cells (3 and 4) were not contained within a single image stack, and were excluded from the whole-cell analysis. For partial volume analysis, grids were 50 nm apart and encompassed a region 1.5 µm either side of the nucleus. Points were classified as being ‘not in cell’ (for cytoplasm at points that did not lie on either mitochondria or intracellular membrane), or as ‘mitochondria’ or ‘intracellular membrane’. A dynamic centre line that accounted for the asymmetry of the cell was used for division into the flattened or rounded hemisphere. To define anterior and posterior hemispheres, we chose the anterior hemisphere to begin where the cuticular portion of the cell first appeared in the images and to end at the centre section of the cell, and the posterior hemisphere to begin at the next section.

Reconstruction of the whole cell was undertaken by manual segmentation. For visualisation in 3D, reconstruction strands of membrane in a section were modelled as representative tubes with a radius of 50 nm, which is the width of the gap between one section and the next. To minimise human error, the model cell was subject to two additional passes through the complete cell. Tomographic reconstructions were checked by a second expert (human) segmenter.

Measurements were taken with IMOD and ImageJ (Schneider et al., 2012). Additional specially developed software tools were used for extraction of data from IMOD models. Statistics were calculated using SPSS (IBM, USA). Data are given as means±s.e.m. Significance was calculated using Student's *t*-tests and one-way ANOVA with Games-Howell post hoc test (where appropriate) or a Kruskal–Wallis test. Images were adjusted for optimal contrast and brightness and assembled into figures using Adobe Photoshop CS6 (Adobe Systems Software Ltd, Ireland).

## Supplementary Material

Supplementary Material
