# Supplementary material for: Association of intracellular and synaptic organization in cochlear inner hair cells revealed by 3D electron microscopy
Source: J Cell Sci. 2015 Jul 15;128(14):2529–40. doi: 10.1242/jcs.170761 (PMC4510854; doi:10.1242/jcs.170761)
Supplement: Supplementary Material [file supp_128_14_2529__index.html]

Supplementary Material 

# Association of intracellular and synaptic organization in cochlear inner hair cells revealed by 3D electron microscopy

## JCS170761 Supplementary Material

- Supplementary Material
