# Supplementary material for: Association of intracellular and synaptic organization in cochlear inner hair cells revealed by 3D electron microscopy
Source: J Cell Sci. 2015 Jul 15;128(14):2529–40. doi: 10.1242/jcs.170761 (PMC4510854; doi:10.1242/jcs.170761)
Supplement: Supplementary Material [file supp_jcs.170761_JCS170761supp.pdf]

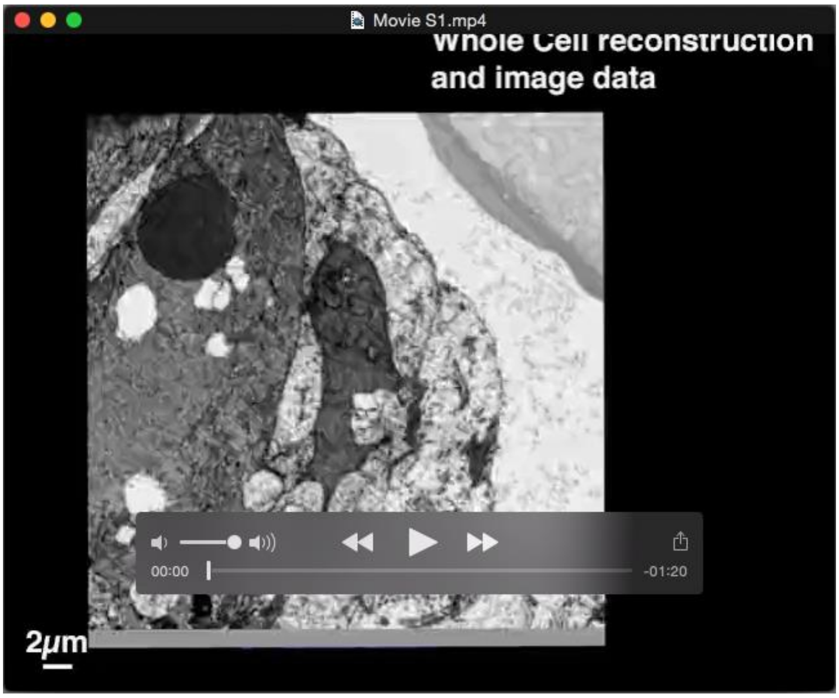

Movie 1. 3D model of Cell 1 reconstructed from SBF-SEM data.
Inner hair cell from a C57/Bl6 mouse. Reconstruction was carried out by manual segmentation. Intracellular structures in the model cell (Cell 1) including mitochondria and membrane types are shown in 3D.

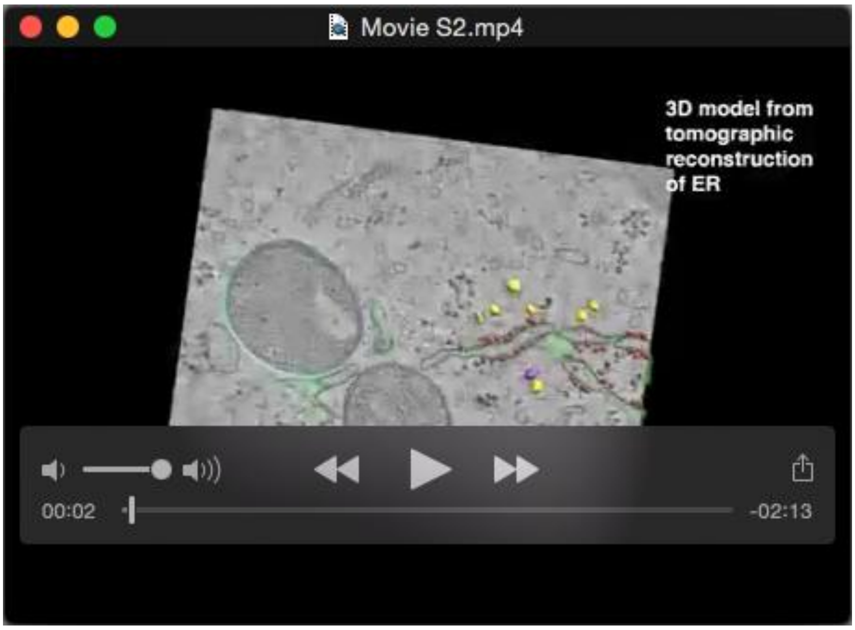

Movie 2. 3D reconstruction from electron tomograms of ER and Synaptic Ribbon.

Intracellular structures from a C57/Bl6 mouse. Reconstruction was carried out by manual segmentation. Mitochondrial and vesicle links are shown at an ER membrane and vesicle-vesicle links are shown at a synaptic ribbon.

Table S1. Comparison of Intracellular membrane distribution, neuronal distribution and mitochondria. Related to Figures 2-4.

Cell	Orientation	Internal Membranes and Mitochondria: Proportion on each side of the cell				Neurons					Mitochondria	
	Orientation of flattened side	Pillar Side (3µm Section)	Modiolar Side (3µm Section)	Pillar Side (Whole Cell)	Modiolar Side (Whole Cell)	Total	Pillar Side	Modiolar Side	Side with highest neuron	Synaptic ribbons (total)	Total	Proportion in association with internal membrane
1	Pillar	74%	26%	68%	32%	17	15	2	Pillar	19	1211	95%
2	Pillar	69%	31%	62%	38%	15	13	2	Pillar	15	940	97%
3	Modiolar	17%	83%	N/A	N/A	N/A	N/A	N/A	N/A	N/A	775	98%
4	Modiolar	27%	73%	N/A	N/A	N/A	N/A	N/A	N/A	N/A	563	98%
5	Pillar	57%	43%	60%	40%	15	11	4	Pillar	15	763	98%
6	Modiolar	30%	70%	37%	63%	14	5	9	Pillar	15	700	98%
7	Modiolar	31%	69%	35%	65%	18	12	6	Pillar	19	754	97%
8	Modiolar	17%	83%	24%	76%	19	8*	10*	Modiolar	19	876	97%
9	Modiolar	35%	65%	41%	59%	17	8	9	Pillar	17	784	97%
10	Modiolar	44%	56%	47%	53%	14	6	8	Pillar	14	848	98%
11	Pillar	66%	34%	47%	53%	N/A	N/A	N/A	N/A	N/A	864	98%

*The centre of one synaptic bouton lay on the central line of the cell and was therefore excluded from the analysis

Table S2. Measurements from intracellular membrane model. Related to Figure 5.

	**Type 1**			**Type 2**	**Type 3**
	**Sheet a**	**Sheet b**	**Sheet c**		
Number of membrane sheets in class	N/A	N/A	N/A	8	2073
Total Surface area of group (μm^2)	9340	1.86 x 10^4	8.68 x 10^4	1.91 x 10^4	1.77 x 10^5
Range (Min-Max) (μm^2)	N/A	N/A	N/A	1460-3570	5.14-865
Mean surface area (μm^2)	N/A	N/A	N/A	2383.8 ±305.5	85.3±2
Proportion of total surface area of all membranes	3.0%	6.0%	27.9%	6.1%	56.9%
Mitochondria density per μm^{-2}	5.46 x 10^{-3}	4.46 x 10^{-3}	3.59 x 10^{-3}	2.52 x 10^{-3}	1.55 x 10^{-3}
